# Effects of *Salmonella enterica* ser. Enteritidis and Heidelberg on host CD4^+^CD25^+^ regulatory T cell suppressive immune responses in chickens

**DOI:** 10.1371/journal.pone.0260280

**Published:** 2021-11-29

**Authors:** Revathi Shanmugasundaram, Keila Acevedo, Mohamad Mortada, Gabriel Akerele, Todd J. Applegate, Michael H. Kogut, Ramesh K. Selvaraj

**Affiliations:** 1 USDA-ARS, Toxicology and Mycotoxins Research Unit, Athens, GA, United States of America; 2 Department of Poultry Sciences, The University of Georgia, Athens, GA, United States of America; 3 U.S. Department of Agriculture-ARS, Plains Area, College Station, TX, United States of America; Universitatsklinikum Erlangen, GERMANY

## Abstract

Poultry infected with *Salmonella* mount an immune response initially, however the immune responses eventually disappear leading the bird to be a carrier of *Salmonella*. The hypothesis of this study is that *Salmonella* infection induces T regulatory cell numbers and cytokine production and suppress host T cells locally in the gut to escape the host immune responses. An experiment was conducted to comparatively analyze the effect of *S*. *enterica* ser. Enteritidis (*S*. Enteritidis) and *S*. *enterica* ser. Heidelberg (*S*. Heidelberg) infection on CD4^+^CD25^+^ T regulatory cell properties in chickens. A total of 144 broiler chicks were randomly distributed into three experimental groups of non-infected control, *S*. Enteritidis infected and *S*. Heidelberg infected groups. Chickens were orally inoculated with PBS (control) or 5x10^6^ CFU/mL of either *S*. Enteritidis or S. Heidelberg at 3 d of age. Each group was replicated in six pens with eight chickens per pen. Chickens infected with *S*. Enteritidis had 6.2, 5.4, and 3.8 log_10_ CFU/g, and chickens infected with *S*. Heidelberg had 7.1, 4.8, and 4.1 log_10_ CFU/g *Salmonella* in the cecal contents at 4, 11, and 32 dpi, respectively. Both *S*. Enteritidis and *S*. Heidelberg were recovered from the liver and spleen 4 dpi. At 4, 11, and 32 dpi, chickens infected with *S*. Enteritidis and *S*. Heidelberg had increased CD4^+^CD25^+^ cell numbers as well as IL-10 mRNA transcription of CD4^+^CD25^+^ cells compared to that in the control group. CD4^+^CD25^+^ cells from *S*. Enteritidis- and *S*. Heidelberg-infected chickens and restimulated with 1 μg antigen *in vitro*, had higher (P < 0.05) IL-10 mRNA transcription than the CD4^+^CD25^+^ cells from the non-infected controls Though at 4dpi, chickens infected with *S*. Enteritidis and *S*. Heidelberg had a significant (P < 0.05) increase in CD4^+^CD25^-^ IL-2, IL-1β, and IFNγ mRNA transcription, the CD4^+^CD25^-^ IL-2, IL-1β, and IFNγ mRNA transcription, were comparable to that in the control group at 11 and 32dpi identifying that the host inflammatory response against *Salmonella* disappears at 11 dpi. It can be concluded that *S*. Enteritidis and *S*. Heidelberg infection at 3 d of age induces a persistent infection through inducing CD4^+^CD25^+^ cells and altering the IL-10 mRNA transcription of CD4^+^CD25^+^ cell numbers and cytokine production in chickens between 3 to 32 dpi allowing chickens to become asymptomatic carriers of *Salmonella* after 18 dpi.

## Introduction

Among the foodborne pathogens of poultry, *Salmonella* is the most important foodborne pathogen of concern to the poultry industry [1]. *Salmonella* infection is caused by *S*. *enterica* colonizing poultry intestines at a very early age and the chickens infected with *Salmonella* after 3 days of age results in persistent infection of poultry with *Salmonella* [2]. *Salmonella* is a facultative intracellular bacterium and induces the host innate inflammatory response, characterized by pro-inflammatory cytokines and granulocyte influx [3]. Inflammatory cytokines increase during the early phase of *Salmonella* infection, and IFNγ is upregulated during a later phase [4]. However, the induced host inflammatory response is ultimately downregulated, leading to *Salmonella* continued survival and persistence in poultry gut for even up to 10 weeks of age [5]. Though *Salmonella* infection induces low levels of mucosal IgA and gut-associated T cell response [6] the induced humoral response does not always translate into protective immune responses in infected chickens [7].

One of the pathways through which *Salmonella* escapes the host immune responses is through inducing T regulatory cell activity, which act to suppress host immune responses directed against commensal bacteria [8]. Gut immune responses facilitate the survival of commensal bacteria by inducing anergy in host immune cells that are directed towards commensal microbes through T regulatory cells. Though *Salmonella* infection causes severe symptoms in humans, chickens infected with *S*. *enterica* do not mount efficient immune responses and are asymptomatic [9], suggesting that *S*. *enterica* is a commensal bacteria in the chicken gut [10]. We earlier identified that *S*. *enterica* serovar Enteritidis (*S*. Enteritidis*)* infection increases T regulatory cell numbers in the chicken gut until 14 dpi [11] to suppress the host immune responses locally in the gut. Identifying if this suppression of host immune responses post-*Salmonella* infection is a local or systemic effect will identify how to address the persistent infection of *Salmonella* in poultry.

Recently, several serovars of *S*. *enterica*, including Heidelberg, Kentucky, Enteritidis, Typhimurium, Montevideo, Senftenberg, and Thompson [12–16] were involved in causing *Salmonella* outbreaks in poultry. The multistate outbreak of multidrug-resistant *S*. *enterica* serovar Heidelberg (*S*. Heidelberg) [17] highlights the importance of studying multiple *Salmonella* serovar infections of chickens. Tight junction proteins like Claudin and Zona occludens are critical components to maintain intestinal barrier function in poultry. *S*. Typhimurium infection decreases tight junction protein mRNA [18] and thereby allowing the pathogen to cross the intestinal lumen and reach blood circulation [19] and spread to the liver, spleen, ovary, and oviduct [20]. T regulatory cell upregulation has been identified to promote gut integrity in experimental cirrhosis model of mice [21]. Therefore, it will be interesting to identify if *Salmonella—*induced T regulatory cells can act to maintain the gut integrity in poultry.

Despite the importance of *Salmonella* as a human pathogen, relatively little is known about how *Salmonella* manages to escape the poultry immune response especially when the birds come into production at 35 d of age. The study hypothesizes that *Salmonella* infection induces CD4^+^CD25^+^ T regulatory cells and suppresses host T cells locally in the gut to escape the host immune responses. The objective of this study is to comparatively analyze the effect of *S*. Enteritidis and *S*. Heidelberg infection on CD4^+^CD25^+^ T regulatory cell properties and CD4^+^CD25^-^ T cells in chickens between 4 to 32 dpi to identify if infection with two different serovars of *Salmonella* can induce host immune cells properties in an antigen-specific pathway. We also studied CD4^+^CD25^+^ T regulatory cell and T cell cytokine profiles (IL-10, TGF-β, IL-1β, LITAF, and IL-2 mRNA) to determine whether the oral infection of *Salmonella* can translocate the intestinal barrier to reach internal organs like the liver and the spleen and induce systemic effect.

## Materials and methods

### Ethics statement

All animal protocols were approved by the Institutional Animal Care and Use Committee at the University of Georgia (AUP: A2017 07-004-Y3-A0). Researchers involved in the *in vivo* trial were trained by the University of Georgia on animal care and handling (UGA IACUC 101 course). Chickens were monitored at least once a day for lethargy, loss of body weight, ruffled feathers, diarrhea, and dehydration during the *in vivo* experiment. Chickens that could not move or refused to eat were immediately humanely euthanized by cervical dislocation. None of the chickens were found dead during this trial no chickens were euthanized for humane reasons. Chickens were euthanized during sampling time points and the last day of the study (day 35).

### Chickens and *S*. Enteritidis and *S*. Heidelberg infection

Wild type *S*. Enteritidis (gift from Dr. G. Rajashekara, The Ohio State University) and Wild type *S*. Heidelberg (gift from Dr. D. Jones, USDA/ARS) were selected on XLT-4 agar for Nalidixic acid resistance at 500 mg L^−1^ of nalidixic acid (Sigma-Aldrich, St. Louis, MO). The nalidixic acid-resistant colonies were grown at 37°C overnight on Muller-Hinton broth containing 500 mg L^−^1 nalidixic acid and further used in experimental studies. Inoculum for infection was prepared from 18 to 24 h cultures. The bacterial concentration was determined spectrophotometrically using a standard curve at a reference wavelength of 600 nm. A stock solution (1x10^9^ CFU/mL) was prepared by diluting the culture in sterile phosphate-buffered saline (pH 7.2).

A total of 144 broiler chicks (Cobb 500, Cobb hatcheries, Cleveland, GA) were randomly distributed into three experimental groups of non-infected control, *S*. Enteritidis infected and *S*. Heidelberg infected groups. All chickens were screened for *Salmonella* by cloacal swab at 0 d of age. Chickens in the infected groups were orally inoculated with 5x10^6^ CFU/mL in 250 μL of either *S*. Enteritidis or S. Heidelberg and chickens in the control groups were inoculated with 250 μL of sterile PBS at 3 d of age. Each group was replicated in six pens with eight chickens per pen. At d 7, 14, 21, 28, and 35 d of age (4, 11, 18, 25, and 32 dpi), one bird per pen (n = 6) from each group was randomly selected and killed by cervical dislocation, and samples were collected.

### Effect of *S*. Enteritidis and *S*. Heidelberg infections on *S*. Enteritidis and *S*. Heidelberg load in the ceca, spleen, and liver

On 4, 11, 18, 25, and 32 dpi, cecal content, spleen, and liver were collected aseptically from six chickens (n = 6) per treatment into stomacher bags, placed on ice, and transported to the laboratory. One g of either cecal content or spleen or liver samples were taken in the stomacher bags and macerated with a pestle. The stomacher bags with macerated samples were mixed with 1X (wt./vol.) of buffered peptone water and the bags were stomached for 90 s. A volume of 10 μl of homogenates was either directly plated or serially diluted in 10^−1^ to 10^−5^ dilutions using the micro dilutions method as described earlier [22]. From every dilution, a volume of 10μl was spotted in triplicate on XLT-4 agar plates. Plates were then incubated for 48 h at 37.5°C. After incubation, colonies were counted and confirmed by SyBr green qPCR using primers described in Table as described earlier [23]. Enumeration data were recorded as CFU/g and then transformed to log10 CFU/g for statistical analysis.

### Effect of *S*. Enteritidis and *S*. Heidelberg infection on CD^+^CD25^+^ cell percentage in the cecal tonsils and spleen

Spleen and cecal tonsils were teased over a 40 μm cell strainer (Sigma-Aldrich, St. Louis, MO) with approximately 5 ml of RPMI to obtain a single-cell suspension. Single-cell suspensions of spleen and cecal tonsils from six chickens per treatment on 4, 11, 18, 25, and 32 dpi (*n* = 6) were concentrated for lymphocyte isolation by density centrifugation utilizing Histopaque (1.077 g/mL; Sigma-Aldrich, St. Louis, MO). Anti-chicken CD25 antibody production was earlier described [24]. Anti-chicken CD25 was labeled with PE using the Lighting-link PE kit (Novus Biologicals, LLC Littleton CO). Cells (1 × 10^6^) were incubated with 10 μg/mL PE linked mouse anti-chicken CD25, 1:200 APC-conjugated mouse anti-chicken CD4 (Southern Biotechnology Associates, Birmingham, AL), and 1:200 dilution of unlabeled mouse IgG for 45 min. The unbound primary antibodies were removed by centrifugation. The percentage of CD4^+^CD25^+^ cells in different organs was analyzed in a flow cytometer (Guava Eascyte; Millipore, Billerica, MA). A total of 50,000 events were collected. CD4^+^CD25^+^ cell percentage was analyzed after gating cells based on forward scatter and side scatter plots for lymphocytes. CD4^+^ and CD4^+^CD25^+^ cell percentage was expressed as a percentage of CD4^+^ cells.

### Effect of *S*. Enteritidis and *S*. Heidelberg infection on immune-related mRNA transcription in CD4^+^CD25^+^ and CD4^+^CD25^-^ cells from cecal tonsils and spleen

Single-cell suspension of cecal tonsils and spleen were labeled for CD4 and CD25 as described above and flow-sorted for CD4^+^CD25^+^ and CD4^+^CD25^-^ cells using an iCyt reflection cell sorter, Champaign, IL (~99% pure) after gating on cells based on forward scatter and side scatter plot for lymphocytes. Total RNA from CD4^+^CD25^+^ and CD4^+^CD25^-^ cells at 4, 11, and 32 dpi were extracted and reverse transcribed into cDNA as described earlier [25] and analyzed for the relative expression of IL-10, TGF-β, IL-1β, LITAF, and IL-2 mRNA transcription, after normalizing for β-actin mRNA transcription, using the primers listed in Table 1. Relative mRNA expression was calculated using the 2^-ΔΔ*Ct*^ method described earlier [26], where Ct is the threshold cycle.

**Table 1:** Primers and PCR conditions for RT qPCR.

Target gene	Sequence (5’-3’)	Annealing Temperature	Reference
IL-10-F	CATGCTGCTGGGCCTGAA	58.0°C	[27]
IL-10-R	CGTCTCCTTGATCTGCTTGATG		
TGF-β4 –F	GACAGCCATCCGCATCTTCT	58.0°C	[28]
TGF-β4 –R	CATACTCCTGGGTCTGGTTGGT		
TLR-4-F	ACCTACCCATCGGACACTTG	60.0°C	[29]
TLR-4-R	TGCCTGAGAGGTCAGGTT		
IL-1β-F	GCATCAAGGGCTACAAGCTC	58.0°C	[30]
IL-1β-R	CAGGCGGTAGAAGATGAAGC		
LITAF-F	ATCCTCACCCCTACCCTGTC	58.0°C	[31]
LITAF-R	GGCGGTCATAGAACAGCACT		
IL-2-F	CTGGGAGAAGTGGTTACTCTGA	59.0°C	[31]
IL-2-R	ACCCGTAAGACTCTTGAGGTTC		
IL-17-F	GCAGATGCTGGATGCCTAAC	55.5°C	
IL-17-R	ATGGAGCCAGTGAGCGTTT		
IFNγ-F	GGCGTGAAGAAGGTGAAAGA	55.4°C	
IFNγ-R	CCTCTGAGACTGGCTCCTTTT		
Claudin-1-F	CATACTCCTGGGTCTGGTTGGT	55.0°C	[32]
Claudin-1-R	GACAGCCATCCGCATCTTCT		
Zona occludens-1-F	TGTAGCCACAGCAAGAGGTG	55.0°C	[33]
Zona occludens-1-R	CTGGAATGGCTCCTTGTGGT		
β-actin–F	ACCGGACTGTTACCAACACC	57.0°C	
β-actin -R	GACTGCTGCTGACACCTTCA		
*S*. Enteritidis-F	GCCGAGCTTGATGACAAACCTG	60.0°C	[34]
*S*. Enteritidis-R	GCGCTTCGCTTTTCCAACTGCC		
*S*. Heidelberg-F	GCCGAGCTTGATGACAAACCTG	55.0°C	[34]
*S*. Heidelberg-R	GCGCTTCGCTTTTCCAACTGCC		

### Effect of *S*. Enteritidis and *S*. Heidelberg infections on tight junction protein mRNA transcription in the jejunum and ceca

On 4, 11, and 32 dpi, approximately 1 cm of jejunum or ceca samples were collected in 2 ml of RNAlater (Qiagen, Germantown, MD) Excess RNAlater was removed from tubes, and samples were stored at -80°C until analyzed. Total RNA was extracted from cecal tonsils and reverse transcribed into cDNA [25] and analyzed for the relative expression of Claudin-1 and Zona occludens-1 mRNA transcription, after normalizing for β-actin mRNA transcription, using the primers listed in Table 1. Relative mRNA expression was calculated using the 2^-ΔΔ*Ct*^ method described earlier [26], where Ct is the threshold cycle.

### Effect of *S*. Enteritidis and *S*. Heidelberg infections on antigen-specific *in vitro* recall response of cecal tonsil CD4^+^CD25^+^ and CD4^+^CD25^-^cells

Heat-killed *S*. Enteritidis and *S*. Heidelberg antigen were prepared by heating 1 mL of 1.25 X 10^8^ *S*. Enteritidis at 65°C for 1 h [35]. Whole cecal tonsils were passed over a 0.4 μm cell strainer to obtain a single-cell suspension. Cecal tonsil cells (1 X 10^8^) were stimulated *in vitro* with 0 or 1 μg of heat-killed *S*. Enteritidis antigen in 1000 μl of RPMI medium supplemented with 5% fetal bovine serum, 1% penicillin plus streptomycin for 48 h. CD4^+^CD25^+^ and CD4^+^CD25^-^ cells from the 48 h culture were flow sorted as described above. The CD4^+^CD25^+^ and CD4^+^CD25^-^ cells cell purities were at least 90% as determined in a flow cytometer. Total RNA was extracted and analyzed for the relative expression of IL-10, IL-2, IFNγ, and IL-17 mRNA as described above.

### Statistical analysis

A one-way ANOVA (JMP software, Cary, NC) was used to examine the effect of different parameters studied on dependent variables. When the effects were significant (*P* < 0.05), differences between means were analyzed by Tukey’s least-square means comparison.

## Results

### Effect of *S*. Enteritidis and *S*. Heidelberg infections on *S*. Enteritidis and *S*. Heidelberg load in the ceca, spleen, and liver

**Fig 1:**
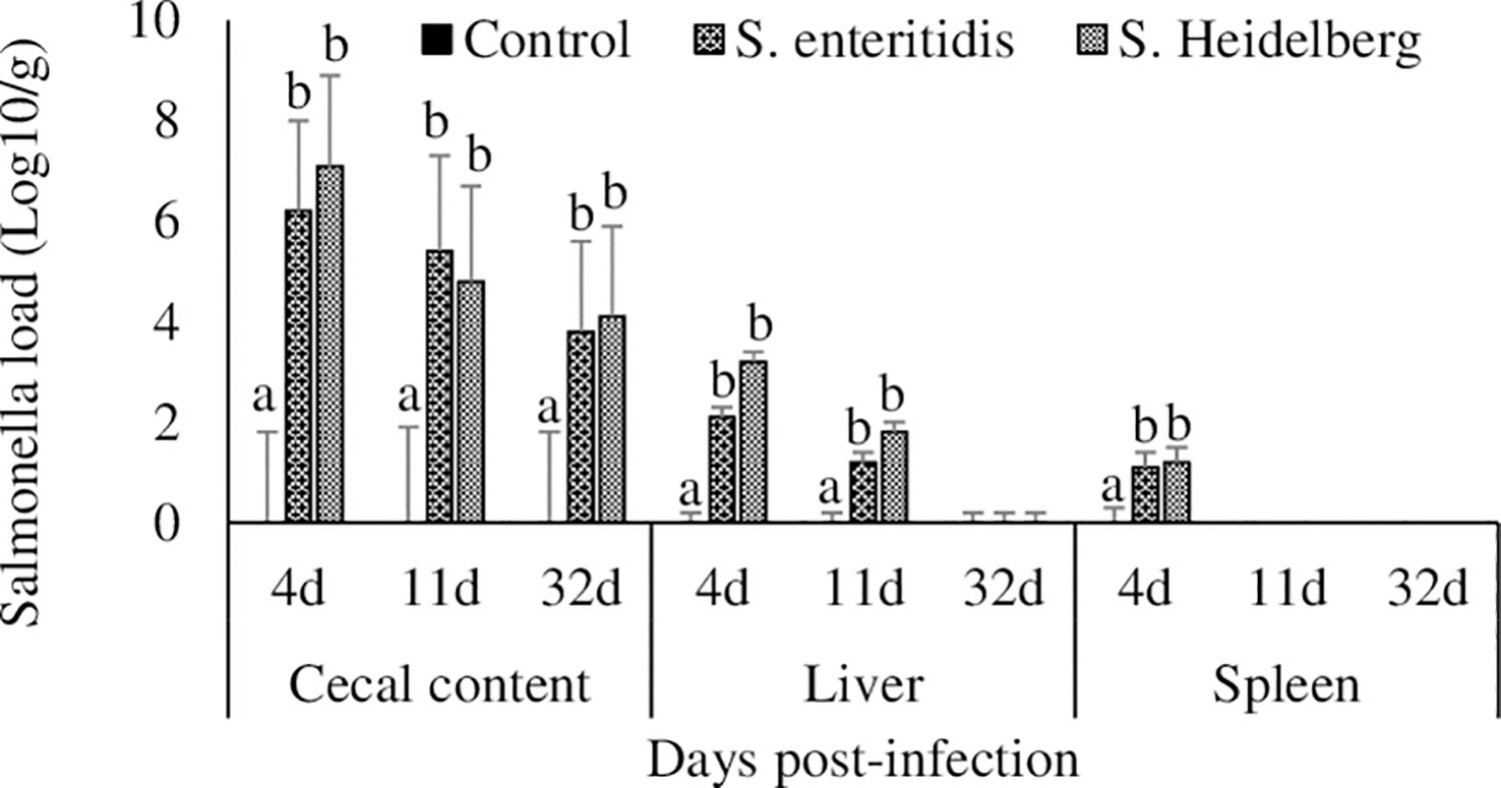
Effect of *S*. Enteritidis and *S*. Heidelberg infections on *S*. Enteritidis and *S*. Heidelberg load in the ceca, spleen, and liver. Chickens (3 d-old) were orally infected with 0 (control) or 5x10^6^ CFU of *S*. Enteritidis or *S*. Heidelberg in six replications. At 4, 11, and 32 d post-infection, *S*. Enteritidis or *S*. Heidelberg loads in the cecal content, liver, and spleen were estimated by a micro-dilution method (CFU/g) and then log-transformed to log10 CFU/g for statistical analysis. Means ± SEM. Bars without a common superscript differ significantly each measured day post-infection (P < 0.05). n = 6.

There were no detectable amounts of *Salmonella* in the cecal content, liver, and spleen of control group chickens at any of the time points studied (Fig 1). Chickens infected with *S*. Enteritidis had 6.2, 5.4, and 3.8 log_10_ CFU/g, and chickens infected with *S*. Heidelberg had 7.1, 4.8, and 4.1 log_10_ CFU/g *Salmonella* in the cecal contents at 4, 11, and 32 dpi, respectively. Both *S*. Enteritidis and *S*. Heidelberg had crossed the intestinal barrier and were recovered from the liver at 4 and 11 dpi. Both *S*. Enteritidis and *S*. Heidelberg were recovered from the spleen at 4 dpi, after which there were no detectable amounts of *S*. Enteritidis and *S*. Heidelberg in the spleen.

### Effect of *S*. Enteritidis and *S*. Heidelberg infections on CD4^+^CD25^+^ cell percentage in the cecal tonsils and spleen

**Fig 2:**
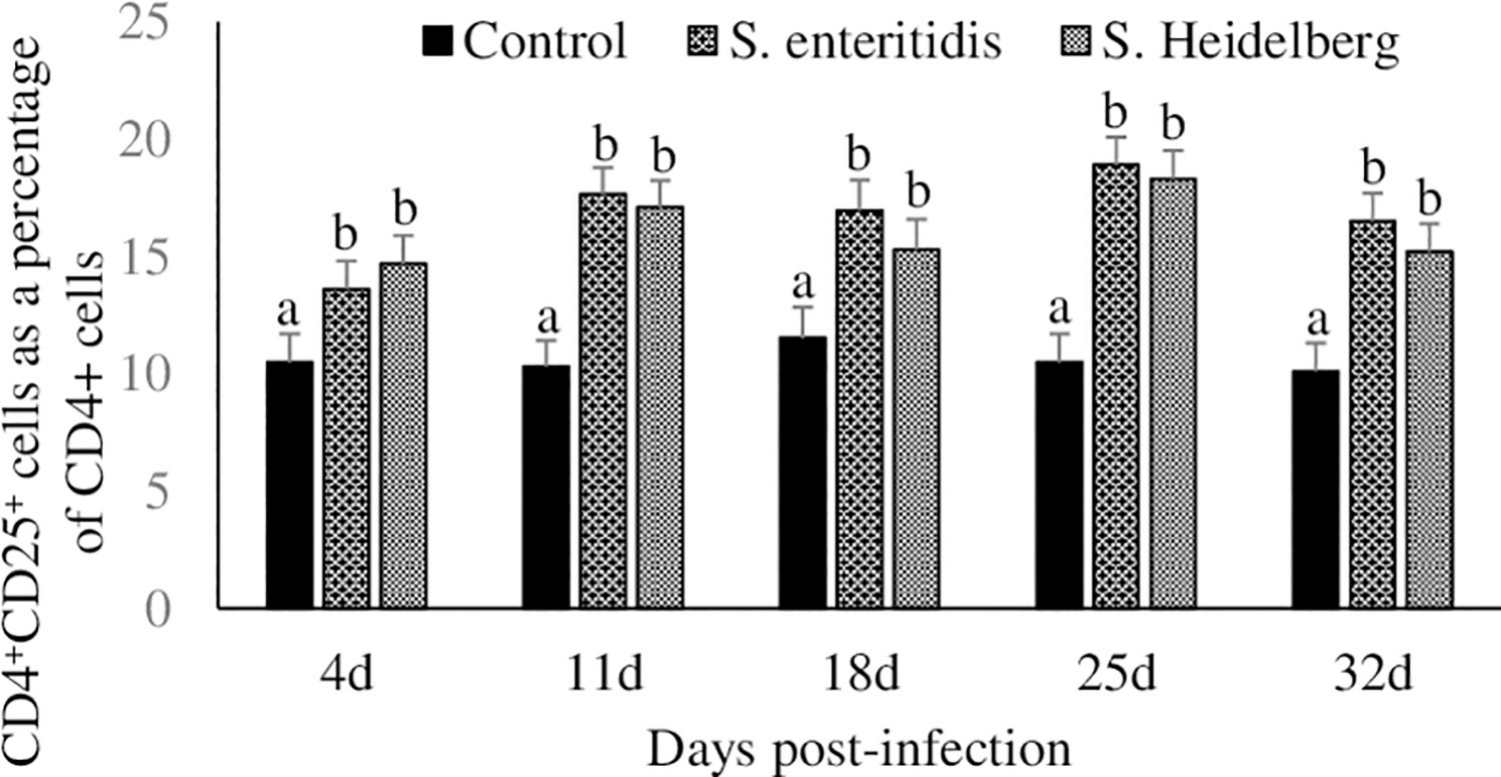
Effect of *S*. Enteritidis and *S*. Heidelberg infections on CD4^+^CD25^+^ cell percentage in the cecal tonsils. Chickens (3 d-old) were orally infected with 0 (control) or 5x10^6^ CFU of *S*. Enteritidis or *S*. Heidelberg in six replications. At 4, 11, 18, 25, and 32 d post-infection, CD4^+^CD25^+^ regulatory T cell percentage in cecal tonsils were analyzed by flow cytometry. CD4^+^CD25^+^ cell percentage was expressed as a percent of CD4^+^ cells to facilitate comparison between samples. Means ± SEM. Bars without a common superscript differ significantly each measured day post-infection (P < 0.05). n = 6.

Approximately 10% of the cecal tonsil CD4^+^ cells were CD4^+^CD25^+^ cells in the control group (Fig 2). Chickens infected with *S*. Enteritidis and *S*. Heidelberg had a significant (P < 0.05) increase in cecal tonsil CD4^+^CD25^+^ cell percentages at 4, 11, 18, 25, and 32 dpi, compared to that in the control group. Chickens in the *S*. Enteritidis infected groups had further 3.1 to 8.4% and chickens in the *S*. Heidelberg infected groups had a further 3.8 to 7.8% increase in cecal tonsil CD4^+^CD25^+^ cell percentages compared to the control group between 4 to 32 dpi.

There were no significant differences (P > 0.05) in the spleen CD4^+^CD25^+^ cell percentages at any of the time point studied.

### Effect of *S*. Enteritidis and *S*. Heidelberg infections on IL-10, TGF-β, IL-1β, LITAF, and IL-2 mRNA transcription in CD4^+^CD25^+^ cells from cecal tonsils and spleen

**Fig 3:**
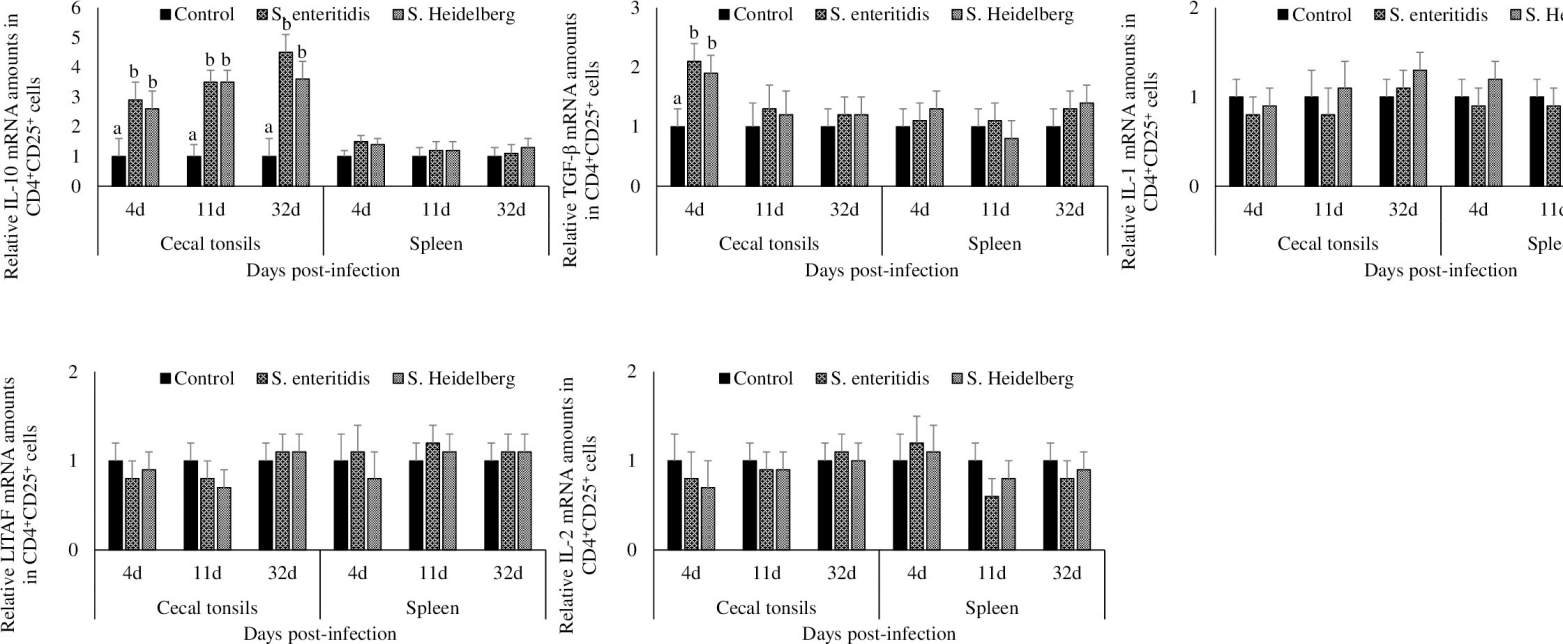
Effect of *S*. Enteritidis and *S*. Heidelberg infections on IL-10, TGF-β, IL-1β, LITAF, and IL-2 mRNA amounts in CD4^+^CD25^+^ cells from cecal tonsils and spleen. Chickens (3 d-old) were orally infected with 0 (control) or 5x10^6^ CFU of *S*. Enteritidis or *S*. Heidelberg in six replications. At 4, 11, and 32 d post-infection, CD4^+^CD25^+^ cells were flow-sorted and analyzed for IL-10, TGF-β, IL-1β, LITAF, and IL-2 mRNA after normalizing for β-actin mRNA. Relative amounts of mRNA were expressed as fold change from the control. Means ± SEM. Bars without a common superscript differ significantly each measured day post-infection (P < 0.05). n = 6.

Chickens infected with *S*. Enteritidis and *S*. Heidelberg had a significant (P < 0.05) increase in the cecal tonsil CD4^+^CD25^+^ cell IL-10 mRNA transcription at 4, 11, and 32 dpi, compared to that in the control group (Fig 3). At 4, 11, and 32 dpi, chickens in the *S*. Enteritidis infected groups had 2.9-, 3.5-, and 4.5- fold, and chickens in the *S*. Heidelberg infected groups had 2.6-, 3.5-, and 3.6- fold increase in cecal tonsil CD4^+^CD25^+^ cell IL-10 mRNA compared to that in the control group, respectively.

Chickens infected with *S*. Enteritidis and *S*. Heidelberg had comparable splenic CD4^+^CD25^+^ cell IL-10 mRNA transcription to that in the control group at all time points studied.

At 4dpi, chickens infected with *S*. Enteritidis and *S*. Heidelberg had a significant (P < 0.05) increase of TGF-β mRNA in the cecal tonsil CD4^+^CD25^+^ cells compared to that in the control group. At 4 dpi, chickens in the *S*. Enteritidis infected groups had 2.1- fold, and chickens in the *S*. Heidelberg infected groups had 1.9- fold increase of TGF-β mRNA in the cecal tonsil CD4^+^CD25^+^ cells compared to that in the control group. At 11 and 32 dpi, chickens infected with *S*. Enteritidis and *S*. Heidelberg had comparable TGF-β mRNA transcription in the cecal tonsil CD4^+^CD25^+^ cells to that in the control group.

There were no significant differences in the cecal tonsil and splenic CD4^+^CD25^+^ cell IL-1β, LITAF, and IL-2 mRNA transcription between the treatment groups at any of the time points studied.

### Effect of *S*. Enteritidis and *S*. Heidelberg infections on antigen-specific *in vitro* recall response of cecal tonsil CD4^+^CD25^+^ and CD4^+^CD25^-^cells

**Fig 4:**
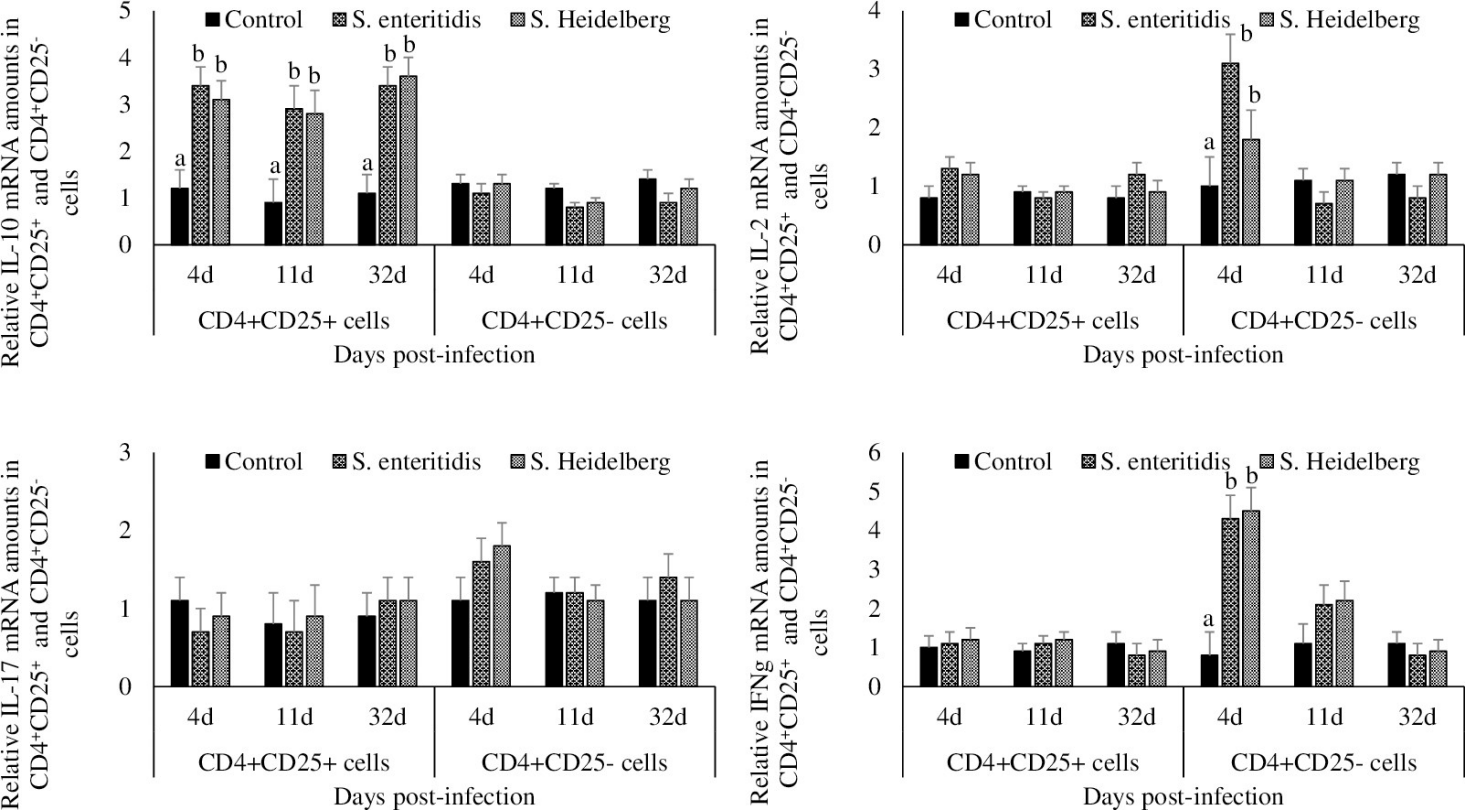
Effect of *S*. Enteritidis and *S*. Heidelberg infections on antigen-specific in vitro recall response of cecal tonsil CD4^+^CD25^+^ and CD4^+^CD25^-^cells. Chickens (3d-old) were orally infected with 0 (control) or 5x10^6^ CFU of *S*. Enteritidis or *S*. Heidelberg in six replications. At 4, 11, and 32 d post-infection, 1 X 10^8^ cecal tonsil cells were stimulated *in vitro* with 0 or 1 μg of heat-killed *S*. Enteritidis or *S*. Heidelberg antigens. Cecal tonsil cells were then incubated with killed antigens from either *S*. Enteritidis or *S*. Heidelberg for 48h. CD4^+^CD25^+^ cells and CD4^+^CD25^-^ cells were flow-sorted and analyzed for IL-10, IL-2 IL-17 and IFN-γ mRNA after normalizing for β-actin mRNA. Relative amounts of mRNA were expressed as fold change from that in the respective 0 μg antigen treated group. Means ± SEM. Bars without a common superscript differ significantly each measured day post-infection (P < 0.05). n = 6.

CD4^+^CD25^+^ cells from *S*. *Enteritidis*- and *S*. Heidelberg-infected chickens and were re-stimulated with 1 μg antigen *in vitro*, had higher IL-10 mRNA content than the CD4^+^CD25^+^ cells from the non-infected controls (P < 0.05; Fig 4).

CD4^+^CD25^-^ cells from *S*. Enteritidis- and *S*. Heidelberg-infected chickens and stimulated with 1 μg antigen *in vitro*, had higher IL-2 and IFN-γ mRNA content than the CD4^+^CD25^-^ cells from the non-infected controls at 4dpi (P < 0.05; Fig 6). There were no significant differences between the IL-2 and IFN-γ mRNA transcription of CD4^+^CD25^-^ cells collected at 11 and 32dpi.

There were no significant differences between the IL-17 mRNA transcription of CD4^+^CD25^+^ cells and CD4^+^CD25^-^ cells collected at 11 and 32dpi.

### Effect of *S*. Enteritidis and *S*. Heidelberg infections on IL-10, TGF-β, IL-1β, LITAF, and IL-2 mRNA transcription in CD4^+^CD25^-^ cells from cecal tonsils and spleen

**Fig 5:**
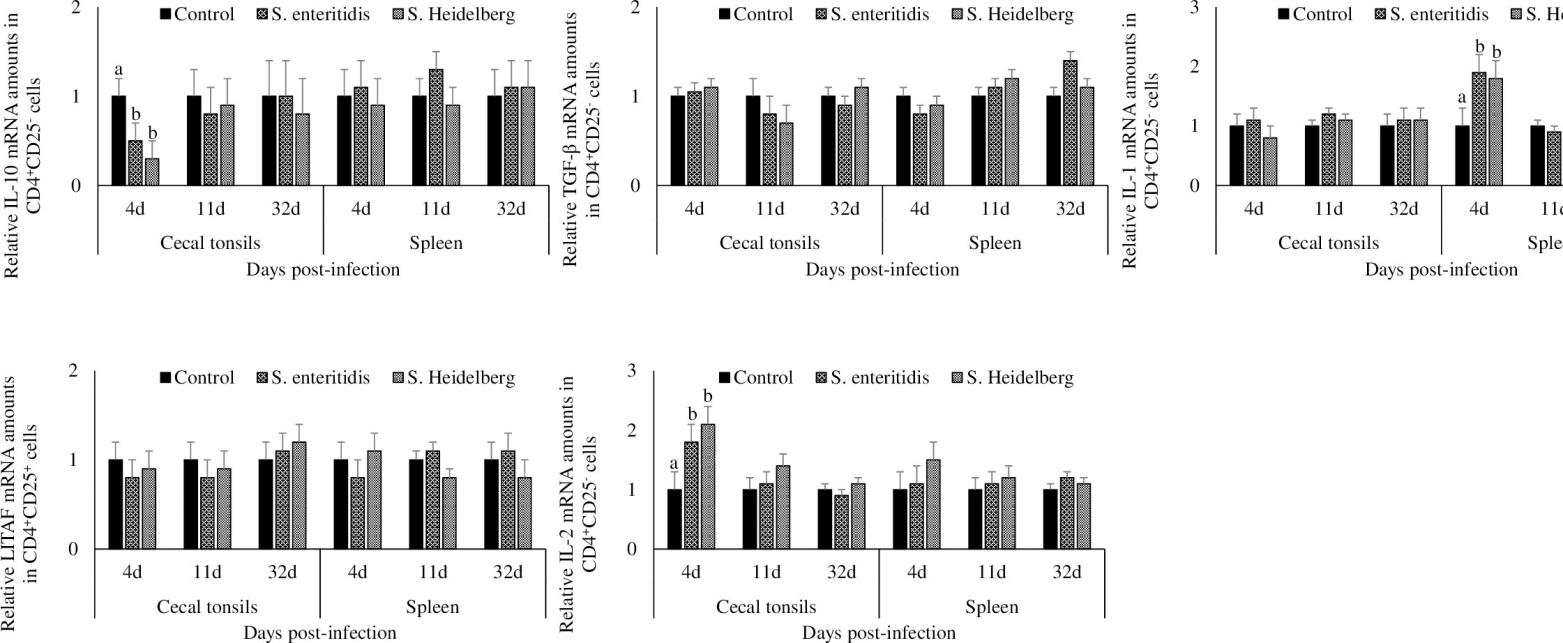
Effect of *S*. Enteritidis and *S*. Heidelberg infections on IL-10, TGF-β, IL-1β, LITAF, and IL-2 mRNA amounts in CD4^+^CD25^-^ cells from cecal tonsils and spleen. Chickens (3 d-old) were orally infected with 0 (control) or 5x10^6^ CFU of *S*. Enteritidis or *S*. Heidelberg in six replications. At 4, 11, and 32 d post-infection, CD4^+^CD25^-^ cells were flow-sorted and analyzed for IL-10, TGF-β, IL-1β, LITAF, and IL-2 mRNA after normalizing for β-actin mRNA. Relative amounts of mRNA were expressed as fold change from the control. Means ± SEM. Bars without a common superscript differ significantly each measured day post-infection (P < 0.05). n = 6.

Chickens infected with *S*. Enteritidis and *S*. Heidelberg had a significant decrease in the cecal tonsil CD4^+^CD25^-^ cell IL-10 mRNA transcription at 4 dpi, compared to that in the control group (P < 0.05; Fig 5). At 4 dpi, chickens in the *S*. Enteritidis infected groups had 50% and chickens in the *S*. Heidelberg infected groups had a 70% decrease in cecal tonsil CD4^+^CD25^-^ cell IL-10 mRNA compared to that in the control group.

At 4 dpi, chickens infected with *S*. Enteritidis and *S*. Heidelberg had a significant increase in the cecal tonsil CD4^+^CD25^-^ cell IL-1β mRNA compared to that in the control group (P < 0.05). At 4 dpi, chickens in the *S*. Enteritidis infected groups had 1.9- fold, and chickens in the *S*. Heidelberg infected groups had a 1.8- fold increase in cecal tonsil CD4^+^CD25^-^ cell IL-1β mRNA compared to that in the control group. At 11 and 32 dpi, chickens infected with *S*. Enteritidis and *S*. Heidelberg had comparable cecal tonsil CD4^+^CD25^-^ cell IL-1β mRNA transcription to that in the control group.

At 4dpi, chickens infected with *S*. Enteritidis and *S*. Heidelberg had a significant increase in the cecal tonsil CD4^+^CD25^-^ cell IL-2 mRNA compared to that in the control group (P < 0.05). At 4 dpi, chickens in the *S*. Enteritidis infected groups had 1.8- fold, and chickens in the *S*. Heidelberg infected groups had 2.1- fold increase in cecal tonsil CD4^+^CD25^-^ cell IL-2 mRNA compared to that in the control group. At 11 and 32 dpi, chickens infected with *S*. Enteritidis and *S*. Heidelberg had comparable cecal tonsil CD4^+^CD25^-^ cell IL-2 mRNA transcription to that in the control group.

There were no significant differences between the cecal tonsil and splenic CD4^+^CD25^-^ cell TGF-β and LITAF mRNA transcription between the treatment groups at any of the time points studied.

### Effect of *S*. Enteritidis and *S*. Heidelberg infections on tight junction protein mRNA transcription in the jejunum and ceca

**Fig 6:**
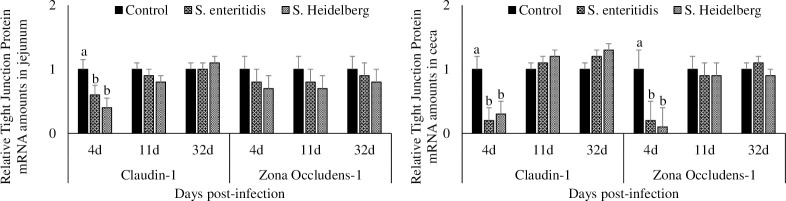
Effect of *S*. Enteritidis and *S*. Heidelberg infections on tight junction protein mRNA amounts in the jejunum and ceca. Chickens (3 d-old) were orally infected with 0 (control) or 5x10^6^ CFU of *S*. Enteritidis or *S*. Heidelberg in six replications. At 4, 11, and 32 d post-infection, jejunal and ceca samples were analyzed for Claudin-1 and Zona occludens-1 mRNA after normalizing for β-actin mRNA. Relative amounts of mRNA were expressed as fold change from the control. Means ± SEM. Bars without a common superscript differ significantly each measured day post-infection (P < 0.05). n = 6.

At 4 dpi, chickens infected with *S*. Enteritidis had a significant 40% decrease and chickens infected with *S*. Heidelberg had a significant 60% decrease in jejunal Claudin-1 mRNA transcription compared to the control group (P < 0.05; Fig 6). At 11 and 32dpi, chickens infected with *S*. Enteritidis and *S*. Heidelberg had comparable jejunal Claudin-1 mRNA transcription with that of the control chickens.

At 4 dpi, chickens infected with *S*. Enteritidis had a 20% decrease, and chickens infected with *S*. Heidelberg had a 30% decrease in jejunal Zona-occludens-1 mRNA transcription compared to the control group (P > 0.05). At 11 and 32 dpi, chickens infected with *S*. Enteritidis and *S*. Heidelberg had comparable jejunal Zona occludens-1 mRNA transcription with that of the control chickens.

At 4 dpi, chickens infected with *S*. Enteritidis had a significant 80% decrease and chickens infected with *S*. Heidelberg had a significant 70% decrease in cecal Claudin-1 mRNA transcription compared to the control group (P < 0.05). At 11 and 32dpi, chickens infected with *S*. Enteritidis and *S*. Heidelberg had comparable cecal Claudin-1 mRNA transcription with that of the control chickens.

At 4 dpi, chickens infected with *S*. Enteritidis had a 80% decrease, and chickens infected with *S*. Heidelberg had a 90% decrease in cecal Zona-occludens-1 mRNA transcription compared to the control group (P < 0.05). At 11 and 32 dpi, chickens infected with *S*. Enteritidis and *S*. Heidelberg had comparable cecal Zona occludens-1 mRNA transcription with that of the control chickens.

## Discussion

This study aimed to evaluate the properties of chicken regulatory T cells and T cells from a gut lymphoid tissue and spleen during *S*. Enteritidis- and *S*. Heidelberg infection. Infecting broiler chickens with *S*. Enteritidis and *S*. Heidelberg at 3 d of age caused *S*. Enteritidis and *S*. Heidelberg colonization of the gut, resulted in *S*. Enteritidis- and *S*. Heidelberg recovery in the liver and spleen, decreased jejunal Claudin-1 and Zona occludens-1 mRNA transcription, increased CD4^+^CD25^-^ regulatory T cell percentages in the cecal tonsils, increased cecal tonsil CD4^+^CD25^+^ regulatory T cell IL-10 mRNA content, decreased cecal tonsil CD4^+^CD25^-^ T cell IL-10 mRNA content and increased IL-10 mRNA production in CD4^+^CD25^+^ restimulated with *Salmonella* antigen.

It has been observed that chickens infected with *S*. Typhimurium at 1-week of age have the persistent infection until 6–7 weeks of age and the persistence has been attributed to the poorly developed immune system of chickens at hatch [2]. This study observed that both *S*. Enteritidis- and *S*. Heidelberg not only persisted in the chicken gut until 35 d of age but also crossed the intestinal barrier and were recovered from both the spleen and the liver. This study also observed that *S*. Enteritidis and *S*. Typhimurium decreases the tight junction protein expression in both the ceca and jejunum. Claudin proteins link adjacent enterocytes and *S*. Typhimurium downregulates the Zona occludens-1 and Claudin-5 mRNA in the ceca of birds to increase translocation of *Salmonella* to the systemic circulation [36]. It has been suggested that the host toll-like receptor-flagellate interactions regulate the ability of *Salmonella* serovars to cross the gut barrier. TLR5-flagellin interactions restrict flagellate serovars like Enteritidis and Typhimurium to the intestine, while permitting non-flagellate *Salmonella* serovars like *S*.Gallinarum and *S*.Pullorum to cross the gut barrier and colonize internal organs [37]. In addition, the Type III secretion system of *Salmonella* facilitates the host signal transduction pathways to decrease tight junction proteins like Zona occludens-1, occludin, and E-cadherin in MDCK-1 cell lines [38] and facilitate *Salmonella* invading the internal organs. It has been suggested that *S*. Typhimurium decreases the tight junction proteins in the jejunum [18] likely contributing to the bacteria translocating from the blood circulation to the internal organs like liver and spleen [19].

*Salmonella* lipopolysaccharide and flagella are pathogen-associated molecular patterns recognized by the host to initiate an immune response that is expected to clear the pathogen [39]. Upon infection with *Salmonella*, innate inflammatory response, characterized by pro-inflammatory cytokines and heterophils proliferation happens within hours [40,41]. This article observed that there was an increase in IL-1β and IFNγ mRNA content of splenic CD4^+^CD25^-^ cells. The observed increase in the IL-1β and IFNγ mRNA of CD4^+^CD25^-^ cells occurred only in the internal organ but not in the cecal tonsils, suggesting that the systemic host immune response to *Salmonella* infection differs from the mucosal immune response to *Salmonella*. IL-17 was not upregulated at any point of the infection with *S*. Enteritidis and *S*. Heidelberg. *S*. Enteritidis has been earlier shown to upregulate IL-17 in the cecal wall at 4 d post-infection, though by 7d post-infection, IL-17 levels are back to normal [42]. This suggests that the effect of *S*. Enteritidis on IL-17 is time dependent.

The interplay between regulatory T cells and other immune cells determines if the outcome of an infection is a successful infection of the host by a pathogen or the successful clearance of the pathogen by the host [43]. T regulatory cells facilitate the survival of commensal bacteria in mammals [44] and intestinal bacteria have co-evolved within the host to stimulate T regulatory cell activity to survive the host gut immune response [45]. Depleting Tregs in mice alters the balance between *Salmonella* proliferation and host immune response and results in clearance of *Salmonella* during a persistent *Salmonella* infection [46]. In this study, we observed that both the numbers of cecal tonsil CD4^+^CD25^+^ cells and the IL-10 mRNA content of the cecal tonsil CD4^+^CD25^+^ cells increased in chickens infected with *S*. Enteritidis- and *S*. Heidelberg. Since IL-10 is a regulatory cytokine that acts to reverse proinflammatory cytokine actions, increased IL-10 transcription of CD4^+^CD25^+^ cells in the gut can be expected to neutralize the increase in pro-inflammatory cytokine produced by other host T cells that will act to clear *S*. Enteritidis- and *S*. Heidelberg infection. This study observed an increase in IL-1β transcription in the spleen of birds infected with *S*. Enteritidis- and *S*. Heidelberg. Increased T regulatory cell numbers and functions can be expected to dampen the immune response against *Salmonella* in the gut leading to *Salmonella* persistence in the chicken gut. Interestingly *S*. Enteritidis- and *S*. Heidelberg infection mediated an increase in the suppressive properties of CD4^+^CD25^+^ cells only in the gut, but not in the spleen; suggesting that *Salmonella*-induced host immune suppression is not systemic but only a localized mucosal response. Though *Salmonella* infection induces macrophage, B and T cell immune response against *Salmonella*, these immune responses eventually wane [47], leading to persistent *Salmonella* infection. Regulatory cells are likely involved in suppressing the host immune response leading to persistent infection of the chicken gut, but not the internal organs.

T regulatory cells in mammalians are characterized by the presence of FoxP3 transcription factor, but functional FoxP3 is yet to be identified in chickens [48]. In the absence of FoxP3, chicken CD4^+^CD25^+^ have been characterized as T regulatory cells [24]. Because CD25 markers are not unique for T regulatory cells, IL-10 mRNA content and ability to suppress naïve T cells using a naïve T cell proliferation suppression assay of CD4^+^CD25^+^ cells are typically used as additional parameters to confirm the T regulatory properties of CD4^+^CD25^+^ cells [24,29]. Our earlier study with *S*. Enteritidis identified that CD4^+^CD25^+^ cells from cecal tonsils of *S*. Enteritidis infected birds had increased IL-10 and suppressed naïve T cells [11]. Though this study analyzed IL-10 mRNA amounts of CD4^+^CD25^+^ cells, but did not analyze the naïve T cell proliferation suppression assay of CD4^+^CD25^+^ cells, the previous study with CD4^+^CD25^+^ cells from *S*. Enteritidis birds, wherein naïve T cell proliferation study was conducted, suggest that CD4^+^CD25^+^ cells are indeed T regulatory cells. It is possible that some of the CD4^+^CD25^+^ cells analyzed in this study are activated T cells or other immune cells.

*S*. Enteritidis- and *S*. Heidelberg mediated increase in CD4^+^CD25^+^ suppressive properties are antigen-specific. *Salmonella* induces iNOS expression to inhibit the proliferation and differentiation of T cells [49] through T regulatory cells as depletion of T regulatory cells abrogated the *S*. Typhimurium induced loss of Th1 response [50]. We observed that the *Salmonella* antigen-induced IL-10 mRNA increase in CD4^+^CD25^+^ cells is antigen-specific as CD4^+^CD25^+^ cells from the control group when restimulated with *Salmonella* antigen did not increase IL-10 production. Earlier it has been observed that *S*. Typhi induces antigen-specific regulatory T cells in humans and migration of antigen-specific regulatory cells to the gut plays an important role in inducing typhoid fever through suppressing antigen-specific T effector cells [51].

This study utilized killed *Salmonella* antigens to stimulate CD4^+^CD25^+^ and CD4^+^CD25^-^ cells *in vitro*. Though recall responses evaluate the ability of the antigens to stimulate different components of T cell signaling pathway, the assay is non-specific in that it cannot distinguish the presence of cross-reactive T-cell responses. Earlier it has been shown that cross priming where peptides from exogenous antigens were presented on class I MHC molecules leading to CD8^+^ T cell stimulation [52]. Another possibility is that the heat-killed antigens used in this study could have stimulated the TLR pathway in a non-antigenic pathway [53] or stimulated the natural killer T cells which can be activated by different bacterial antigens and lipopolysaccharide or flagellin [54].

It can be concluded that *S*. Enteritidis and *S*. Heidelberg infection at 3 d of age induces a persistent infection through induction of T regulatory cells. Altering the IL-10 mRNA transcription of T regulatory cells in chickens between 3 to 32 dpi and chickens become asymptomatic carriers of *Salmonella* after 18 dpi. The upregulation of T regulatory cell IL-10 cytokine production by *Salmonella* was antigen-specific and the immune response was observed only in the gut where *Salmonella* had colonized until 32 dpi. *Salmonella* infection of chickens induced T cell IL-2 and IL-1β transcription at 3 dpi, however there were no differences in the studied cytokine transcription of T cells between the control group and *Salmonella* infected groups after 11 dpi.
